# Antidiabetic Effects of Hydroxytyrosol: In Vitro and In Vivo Evidence

**DOI:** 10.3390/antiox8060188

**Published:** 2019-06-21

**Authors:** Filip Vlavcheski, Mariah Young, Evangelia Tsiani

**Affiliations:** 1 Department of Health Sciences, Brock University, St. Catharines, ON L2S 3A1, Canada; fvlavcheski@brocku.ca (F.V.); my14ac@brocku.ca (M.Y.); 2 Centre for Bone and Muscle Health, Brock University, St. Catharines, ON L2S 3A1, Canada

**Keywords:** insulin resistance, diabetes, hydroxytyrosol, olive oil, phenols, antioxidant, antidiabetic

## Abstract

Insulin resistance, a pathological condition characterized by defects in insulin action leads to the development of Type 2 diabetes mellitus (T2DM), a disease which is currently on the rise that pose an enormous economic burden to healthcare systems worldwide. The current treatment and prevention strategies are considerably lacking in number and efficacy and therefore new targeted therapies and preventative strategies are urgently needed. Plant-derived chemicals such as metformin, derived from the French lilac, have been used to treat/manage insulin resistance and T2DM. Other plant-derived chemicals which are not yet discovered, may have superior properties to prevent and manage T2DM and thus research into this area is highly justifiable. Hydroxytyrosol is a phenolic phytochemical found in olive leaves and olive oil reported to have antioxidant, anti-inflammatory, anticancer and antidiabetic properties. The present review summarizes the current in vitro and in vivo studies examining the antidiabetic properties of hydroxytyrosol and investigating the mechanisms of its action.

## 1. Introduction

**Figure 1:**
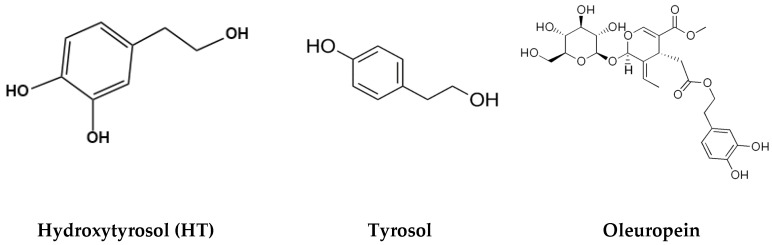
Chemical structures of hydroxytyrosol, tyrosol, and oleuropein.

The composition of the culturally-based diet of the Greeks, Spanish, and Italians was first connected to cardiovascular health and lower cholesterol levels by American researcher Ancel Keys nearly fifty years ago [1,2,3,4,5]. This was termed the “Mediterranean diet” and has since been studied extensively, with research showing clear beneficial effects on health and lifespan. The impact of this type of diet is especially relevant to the studies of metabolic disease, cardiovascular well-being and cancer [5,6,7,8,9]. The core “Mediterranean triad” encompasses food derived from wheat, grapes, and olives [10]. As the principal source of lipids in the Mediterranean diet, olive oil has attracted the attention of many scientists attempting to determine how its chemical constituents affect the body. Fatty acids make up most of the mass of olive oil, but it also contains up to 1 gram per kilogram of phenolic compounds [11]. While thirty different phenols have been identified in olive oil, the ones with the highest relative abundances are hydroxytyrosol, tyrosol, and oleuropein (Figure 1). Hydroxytyrosol, often abbreviated as HT, and tyrosol are metabolic derivatives of oleuropein following hydrolysis and are structurally similar [11,12].

Hydroxytyrosol (HT) is the phenol present in the highest concentration in olive oil (Table 1) and is also a product of oleuropein metabolism in the body [12,13]. Much research has been conducted over the past twenty years attempting to determine the health effects of oleuropein [14], hydroxytyrosol, tyrosol, and other phenolic compounds found in olive oil [9,11,15,16,17]. Evidence shows that hydroxytyrosol has antioxidant properties, affects glucose and lipid homeostasis and may protect against diabetes [17,18,19,20,21]. In the present review, the relevant in vitro and in vivo studies examining the antidiabetic effects of HT are summarized.

Currently, pharmacological agents used for the treatment of T2DM include drugs that increase peripheral (muscle and fat) glucose transport while decreasing hepatic glucose production by inhibiting gluconeogenesis (biguanides and thiazolidinediones (TZDs)) (Table 2). Metformin, a biguanide, is the first line of treatment for T2DM. However, its use is associated with an increased risk of lactic acidosis and gastrointestinal (GI) disturbances including cramping, nausea, vomiting, and diarrhea [23,24]. The use of TZDs (rosiglitazone and rioglitazone) is associated with bladder cancer, heart failure, hepatitis, and weight gain. Additionally, agents that stimulate insulin release from the pancreatic β-cells, sulfonylureas (glyburide and glipizide glimepiride) and meglitinides (repaglinide), are routinely prescribed but are associated with a high risk of hypoglycemia. Another class of medication, usually prescribed to prediabetics, is intestinal glucose absorption inhibitors that act by hindering α-glucosidase enzymes and, thereby, absorption of glucose (acarbose, voglibose, and miglitol). However, these drugs are not very potent and their use in North America is quite low. In the last decade, several new therapies for T2DM have become available. Gliptins and dipeptidyl peptide 4 (DDP-4) inhibitors (sitagliptin, saxagliptin, vidagliptin, linagliptin, and alogliptin) hinder glucagon release, thereby reducing blood glucose levels. However, significant side effects may occur including heart failure, pancreatitis, and pancreatic and prostate cancer [25]. Similarly, glucagon-like peptide 1 (GLP-1) receptor agonists (liraglutide, exenatide, and dulaglutide) inhibit glucagon release and stimulate insulin production, consequently lowering blood glucose levels. GLP-1 receptor agonists are associated with a lower risk of hypoglycemia in comparison to sulfonylureas and meglitinides. However, the mechanism of action is similar to the DPP-4 inhibitors. As a result, potential life-threatening conditions such as pancreatic cancer, pancreatitis and heart failure may occur [26]. Sodium–glucose cotransporter 2 (SGLT2) inhibitors (canagliflozin, capagliflozin) are the most recent antidiabetic drugs currently available and they exhibit glucose lowering properties by inhibiting renal glucose reabsorption and stimulating glucose excretion. Although initially promising, these drugs are also associated with adverse health effects including severe hypotension, urinary tract infections (UTI), and ketoacidosis [27,28].

The risks associated with existing T2DM treatments emphasize a continued need to develop more effective T2DM therapeutics with fewer side effects. Despite the progress in the last decade in terms of available treatment/management approaches, T2DM is a disease currently on the rise and poses an enormous burden to our health care systems globally. In addition, approximately 50% of the T2DM-affected population are living in poverty-stricken areas in Africa and Asia. Therefore, there is an urgent need not only for more effective but also for affordable treatment options. Novel compounds, that exhibit insulin-like effects, improve insulin sensitivity, enhance the efficacy of already existing antidiabetic agents and have very few side effects, are greatly desired as they will broaden the spectrum of treatment options for insulin resistance and T2DM.

## 2. In Vitro Evidence: Antidiabetic Effects of Hydroxytyrosol (HT)

### 2.1. Effects of Hydroxytyrosol (HT) on Skeletal Muscle Cells

Treatment of C2C12 cells with HT (1–50 μM) increased creatine kinase activity and myosin heavy chain expression, which are indicators of muscle cell differentiation and strength of contraction, respectively, therefore demonstrating a possible improvement in muscle adaptation to exercise by HT (Table 3) [29]. In addition, treatment with HT attenuated the tumor necrosis factor-α (TNF-α)-induced downregulation of mitochondrial biogenesis by increasing peroxisome proliferator-activated receptor-gamma coactivator (PGC)-1α, mitochondrial complexes (I and II) and myogenin expression. This indicated that HT improves mitochondrial development and function in muscle cells under inflammatory stress [29]. In another study, treatment of C2C12 myoblasts with HT (5–20 μM) resulted in attenuation of H_2_O_2_-induced apoptosis and oxidative stress [30]. Additionally, HT completely prevented the H_2_O_2_-induced morphological changes, swollen mitochondria and presence of autophagic vacuoles [30]. Drira and Sakamoto (2013) found that exposure of C2C12 myotubes to hydroxytyrosol-acetate (12 h at 25–75 µM), which acts similarly to HT, resulted in significantly increased glucose uptake in a dose-dependent manner [31]. Another study found that treatment of C2C12 myotubes with hydroxytyrosol-acetate (1–50 µM for 24h) significantly attenuated the *tert*-butylhydroperoxide (t-BHP)-induced mitochondrial damage, optic atrophy 1 (OPA-1) cleavage and muscle degradation while increasing oxygen consumption capacity, ATP production, activities of mitochondrial complex I, II and V, and myosin heavy chain expression [32]. Additionally, the cell viability was markedly increased by HT treatment (Table 3) [32]. Altogether, the study indicates an effect of HT to protect against oxidative stress-induced skeletal muscle damage. 

The studies above show that HT has the potential to affect key signaling molecules involved in mitochondrial function and development, protect against oxidative stress-induced muscle damage [29,30,32] and significantly increase muscle cell glucose uptake [31]. These studies indicate that HT could have potential antidiabetic effects on muscle tissue directly and further studies are required to investigate this assumption.

### 2.2. Effects of Hydroxytyrosol (HT) on Adipocytes

In one study, exposure of 3T3-L1 adipocytes to HT (0.1-10 µM) resulted in enhanced mitochondrial biogenesis and oxygen uptake (Table 4) [33]. Markers of mitochondrial capacity including peroxisome proliferator-activated receptor γ (PPARγ), PPARγ coactivator (PGC)-1alpha (PGC1α), nuclear respiration factor 1 and 2 (NRF1 and NRF2), mitochondrial transcription factor A and mitochondrial complexes I, II, III and V were significantly upregulated. In addition, to the upregulation of mitochondria number and mtDNA, intracellular fatty acids were reduced and oxygen consumption was increased. More importantly, HT significantly increased the phosphorylation of the energy sensor AMP-activated protein kinase (AMPK) and its downstream target acetyl-CoA carboxylase (ACC). This finding indicates that activating AMPK may be the possible mechanism for HT-induced expression of PGC1α leading to mitochondrial biogenesis [33]. Exposure of mouse-derived C3H10 T1/T2 pre-adipocytes to HT (0.5–25 µM for 4 or 7 days) significantly inhibited adipocyte differentiation and lipid accumulation [34]. Fat droplet size and number were dose-dependently decreased and hormone-sensitive lipase (HSL) was downregulated by HT treatment. Furthermore, HT inhibited rosiglitazone-stimulated lipid synthesis shown by a decrease in carnitine palmitoyltransferase I (CPT1β) and downregulation of adipogenesis regulators such as PPARγ/α, CCAAT-enhancer-binding proteins (C/EBPα) and differentiation markers aP2 (adipocyte fatty acid binding protein) and adiponectin [34]. Interestingly, GLUT4 gene expression was also downregulated most likely as a consequence of HT inhibiting the two master regulators PPARγ and C/EBPα. Altogether, these studies present evidence showing HT suppressing adipogenesis and lipogenesis in adipocytes [34], however the effects on glucose uptake remain to be investigated. Treatment of 3T3-L1 preadipocytes with HT (100–150 µM) resulted in dose-dependent inhibition of cell division during mitotic clonal expansion thus causing cell cycle delay and increased lipid accumulation with no effect on cell viability [35]. Additionally, adipogenesis related genes were significantly downregulated including PPARγ, sterol regulatory element-binding transcription factor 1 (SREBF1), C/EBPα and their downstream target genes GLUT4, cluster of differentiation 36 (CD36) and fatty acid synthase (FAS) [35]. Similar effects were seen when 3T3-L1 adipocytes were treated with HT-acetate (25–75 µM) [31]. A significant inhibition of adipogenesis and lipid accumulation was seen with HT-acetate which was associated with downregulation of PPARγ, SREBP-1c, C/EBPα, GLUT4, CD36, and FAS [31]. Increased lipolysis was also indicated via increase of the quantity of glycerol being released and the activation of hormone-sensitive lipase (HSL) [31]. Exposure of 3T3-L1 adipocytes to HT (0-150 µM) resulted in a dose-dependent increase in lipolysis and glycerol release while decreasing adipocyte triglyceride accumulation [36]. The phosphorylation of proteins involved in lipolysis pathways, including hormone-sensitive lipase (HSL), ERK and perilipin, were significantly increased [36]. Overall expression of HSL, adipocyte triglyceride lipase (ATGL) and adipogenesis proteins PPARγ and C/EBPα were decreased. Despite the negative effect of HT on the expression levels of HSL and ATGL the phosphorylation and therefore activation of HSL and lipolysis was enhanced. The decrease in expression levels may be due to a compensatory mechanism or negative feedback loop resulting from the increased HSL activation. Furthermore, pretreatment with PKA and ERK1/2 inhibitor attenuated the HT-stimulated lipolysis, indicating that PKA and ERK may be involved in the HT-induced lipolysis [36]. Exposure of 3T3-L1 and human Simpson-Golabi-Behmel syndrome (SGBS) adipocytes to HT (0.1–20 µM) prevented the TNFα-induced suppression of total adiponectin secretion and protein expression [37]. Additionally, HT prevented the TNF-α-induced downregulation of PPARγ and JNK phosphorylation. This study showed that the deleterious effects of TNFα are attenuated by HT [37]. Treatment of 3T3-L1 preadipocytes with HT (10–100 µM) resulted in inhibition of cell differentiation, increased FAS and lipoprotein lipase (LPS) gene expression while simultaneously downregulating PPARγ and cannabinoid receptor type 1 (CB1) gene expression [38]. Recent studies on obesity emphasize the importance of discovering food components that have the ability to suppress adipocyte proliferation and differentiation and thus adipose tissue expansion. A new proposed approach includes molecules that may potentially modulate endocannabinoid receptor gene expression [39]. Another study showed that exposure of human primary visceral preadipocytes to HT (5–70 µg/mL) resulted in decreased triglyceride accumulation and increased apoptosis, lipolysis, glycerol release and expression of adipogenesis inhibiting genes such as GATA- binding factor 2 and 3 (GATA2 and GATA3) [40], protein Wnt-3A (WNT3A), secreted frizzled-related protein 5 (SFRP5), hairy and enhancer of split-1 (HES1), and NAD-dependent deacetylase sirtuin-1 (SIRT1). Additionally, genes involved in promoting adipogenesis including leptin (LEP), sterol regulatory element-binding protein 1 (SREBF1), Cyclin D1 (CCND1) and fibroblast growth factor 1 (FGF1) were significantly downregulated [40]. On the other hand, Anter et al. 2016 showed that exposure of human bone marrow mesenchymal stem cells (MSCs) to HT (100 µM) as they differentiated into adipocytes, resulted inupregulated adipocyte differentiation genes such as PPARγ and fatty-acid binding protein 4 (FABP4) as well as fat vesicle formation in the MSC adipocytes. Additionally, lipid protein lipase (LPL) gene expression was modestly but non-significantly decreased (Table 4) [41].

The above studies suggest that HT decreases adipocyte differentiation and proliferation [31,35,36,38,40] and reduces the number and size of adipocyte lipid droplets [34]. Additionally, HT significantly downregulates the genes involved in adipogenesis and obesity while exhibiting substantial anti-inflammatory properties [31,33,34,35,36,37,38]. Furthermore, these studies show significant increase in lipolysis and related enzymes such as HSL and ATGL [31,36,40]. It is important to note that the increase in adipocyte lipolysis in vitro may suggest antidiabetic properties by decreasing the size/expansion of adipocytes, however an upsurge in lipolysis in vivo may significantly increase circulating FFA levels leading to hyperlipidemia, lipid toxicity and exacerbation of insulin resistance and T2DM symptoms. Therefore, more in vivo studies investigating the effect of HT on adipose tissue and circulating lipid levels are needed.

### 2.3. Effects of Hydroxytyrosol (HT) on Hepatocytes

Treatment of primary cultured mouse hepatocytes with HT (100 µM) in conditions mimicking ischemia/reperfusion (I/R) resulted in a dose-dependent decrease in apoptosis and increased cell viability (Table 5) [42]. Additionally, HT significantly ameliorated the I/R-induced decrease in antioxidant enzymes such as SOD1, SOD2 and catalase (CAT). Overall, treatment with HT protected against I/R-induced injury and oxidative damage in mouse hepatocytes [42]. Treatment of rat hepatocytes with HT (25 µM) resulted in reduced de novo lipid synthesis including fatty acid, triglyceride and cholesterol without affecting cell viability [43]. Additionally, exposure to HT significantly decreased the activity of crucial enzymes involved in fatty acid synthesis (ACC), triglyceride synthesis (diacylglycerol acyltransferase) and cholesterogenesis (3-hydroxy-3-methyl-glutaryl-CoA reductase) [43]. Moreover, HT increased the phosphorylation of AMPK and its downstream target ACC suggesting that the mechanism of reduced lipid synthesis by HT is AMPK-mediated [43]. These findings indicate that HT decreases lipid synthesis in hepatic tissue. Another study showed that exposure of vitamin E-deficient rat liver microsomes to HT (0.05–2 mM) decreased the formation of thiobarbituric acid reactive substances (TBARS), a biomarker of lipid peroxidation, and thereby decreased lipid peroxidation [44]. TBARS are formed when malonaldehyde, a product of lipid oxidation reacts with thiobarbituric acid leading to cell damage [45]. In a more recent study, exposure of vitamin E-deficient rat liver microsomes to modified HT compounds (0.05–2 mM) resulted in a stronger, more potent inhibition of harmful lipid peroxidation and TBARS formation compared to pure HT (Table 5) [46]. 

Overall, the studies above have shown that HT exhibits hepatoprotective properties by decreasing apoptosis, increasing antioxidant activity and hepatocyte viability [42,44,46]. More importantly, treatment with HT significantly reduced lipid synthesis [43]. Accumulation of lipids in hepatocytes is often associated with the development of hepatic steatosis which subsequently leads to lower glucose utilization, liver injury and fibrosis thus exacerbating the development of insulin resistance and T2DM. These studies indicate that HT may protect against hepatic steatosis and the development of hepatic insulin resistance.

### 2.4. Effects of Hydroxytyrosol (HT) on Pancreatic Cells

Treatment of rat pancreatic tissue with HT (50 µg/mL) attenuated the hyperglycemia-induced decline in insulin secretion (Table 6) [47]. This indicates that HT may enhance pancreatic insulin secretion in hyperglycemic conditions. In contrast to these finding, exposure of INS-1β cells to HT (0.1–30 µM) did not relieve the hyperglycemia-induced decline in insulin secretion [48]. However, 3-hydroxytyrosol (3-HT) significantly inhibited the formation and cytotoxicity of amylin aggregates in INS-1 β cells. The estimated HT concentration for half maximal inhibition (IC_50_) of amylin aggregates formation was 100 µM [48]. These findings suggest that HT may have a dose-dependent remodeling and inhibitory effect on pancreatic amylin aggregates, which occur frequently in T2DM. Amyloid aggregates contribute to endoplasmic reticulum (ER) stress, mitochondrial damage and membrane disruption leading to β cell death and thereby T2DM [49,50,51]. One of the characteristics of T2DM is progressive deficit in β cell function and mass with increased apoptosis. Therefore preventing the progression of amyloid aggregates may protect the β cells from death (Table 6) [52]. Additionally, the formation of amyloids is often seen in several neurodegenerative diseases such as Huntington’s, Alzheimer’s and Parkinson’s where similarly to T2DM, accumulation of locally expressed misfolded proteins share the tendency to produce amyloid aggregates [51]. 

Experiments with hydroxytyrosol (HT) exposure of skeletal muscle cells, adipocytes, hepatocytes, and pancreatic cells indicate that HT may have effects that could be beneficial in the treatment of diabetes or metabolic syndrome. The few studies examining the effects of HT on skeletal muscle cells have found that HT may increase oxidative capacity and muscular health by supporting mitochondrial biogenesis and protecting myocytes from oxidative stress, in addition to increasing glucose uptake [29,30,31,32]. Multiple studies exposing adipocytes to HT report diminished adipogenesis, enhanced mitochondrial capacity, and decreased lipid accumulation with only one study reporting increased adipocyte differentiation [31,33,34,35,36,37,38,40,41]. Most cell culture studies of hepatocytes suggest that HT attenuates oxidative stress in the liver, with one reported instance of reduced lipid synthesis [42,43,44,46,53,54]. The impact of exposure of pancreatic cells to HT has not been established, but preliminary research indicates that it may enhance insulin secretion and inhibit amylin amyloid β cell damage [47,48].

## 3. In Vivo Evidence: Antidiabetic Effect of Hydroxytyrosol

### 3.1. Effect of Hydroxytyrosol (HT) on Alloxan-Induced Diabetes in Rodents

Hamden et al. induced diabetes in male Wistar rats via intraperitoneal injections of alloxan monohydrate (150 mg/kg), and animals with hyperglycemia (blood glucose levels of 2 g/L after 2 weeks) were retained for experimentation (Table 7) [47]. The treatment groups were given daily intraperitoneal injections of olive mill waste monomeric phenols (F1), olive mill waste polymeric phenols (F2), or purified hydroxytyrosol (F3) at 20 mg/kg for two months. All three treatments, especially purified HT (F3), resulted in significantly decreased blood glucose levels. The hepatic toxicity indicators TBARS, bilirubin, and fatty cysts were reduced in animals receiving HT treatment. Hepatic glycogen, circulating high-density lipoprotein (HDL), and antioxidant enzymes (SOD, CAT, and GPX) in the liver and kidney were increased by HT. Additionally, treatment with HT attenuated the deleterious effects of alloxan in pancreatic β cells [47]. Jemai et al. (2009) examined the possible antidiabetic and antioxidant benefits of oleuropein and HT administration in male Wistar rats. Diabetes was induced with an intraperitoneal injection of alloxan (180 mg/kg) preceding treatment for 4 weeks with HT dissolved in drinking water to reach concentrations of 8 or 16 mg/kg [55]. All treatment groups showed inhibition of hyperglycemia, hypercholesterolemia, and hepatic oxidative damage (TBARS) with simultaneous increases in hepatic glycogen and antioxidant enzymes (SOD, CAT). The 16 mg/kg dose had stronger effects than the 8 mg/kg dose, showing dose-dependence [55]. This study showed the ability of HT to retain healthier lipid profiles, glucose levels, and antioxidant activity in a diabetic rat model. 

### 3.2. Effect of Hydroxytyrosol (HT) on Streptozotocin-Induced Diabetes in Rodents

Hamden et al. (2010) used intraperitoneal streptozotocin (STZ) or STZ (150 mg/kg) and nicotinamide (1000 mg/kg) injections to induce diabetes in male Wistar rats (Table 7) [56]. The rats that displayed a moderate diabetic phenotype with hyperglycemia after 2 weeks were treated with 20 mg/kg HT for two months before serum and tissues from the pancreas and small intestine were isolated. Treatment with HT significantly lowered blood glucose, low-density lipoprotein (LDL)-cholesterol and plasma triglycerides while HDL-cholesterol was increased. Additionally, HT reduced the STZ-induced increase of intestinal enzymes (maltase, lactase and sucrose) that are often elevated in diabetes. These enzymes are imperative for the digestion of disaccharides into simple glucose which is readily available for intestinal absorption [57] and therefore the increased number and activities of the enzymes may lead to hyperglycemia, a major characteristic of diabetes. Furthermore, HT treatment inhibited intestinal lipase and consequently, decreased lipid absorption [56]. In the pancreas, HT treatment increased the activities of antioxidant enzymes SOD, CAT, GSH, and GPX and decreased the formation of harmful advanced glycate end-products (AGE). Pancreatic cells showed lower levels of TBARS and LDH (lactate dehydrogenase) activity [56]. Overall, HT had a hypoglycemic and hypolipidemic effect on diabetic rats while providing protection from oxidative damage (Table 7). Ristagno et al. (2012) also induced diabetes with an intraperitoneal injection of STZ (60 mg/kg) in male Sprague-Dawley rats before administering HT daily by intragastric gavage in doses of either 10 or 100 mg/kg for 6 weeks [58]. HT was found to inhibit the hyperglycemia-induced increases in plasma TBARS and also prevented impairments in nerve conduction velocity (NCV), thermal nociception, and Na^+^/K^+^-ATPase activity [58]. This study showed that HT could mitigate peripheral neuropathy caused by diabetes. Male Wistar rats were given oral HT daily at doses of 0.5, 1, 2.5, 5, or 10 mg/kg for 7 days before diabetes was induced with 50 mg/kg injected STZ in the study by López-Villodres et al. (2016) [59]. HT treatment was continued for two months after the induction of diabetes. Treatment lowered oxidative and nitrosative stresses, inflammatory markers, platelet aggregation, and aortic wall area in comparison to the non-treated diabetic group [59]. It was concluded that HT may attenuate the vasculopathy or blood vessel inflammation induced by diabetes. Reyes et al. (2017) administered HT to male Wistar rats via gavage at dosages of 1, 5 or 10 mg/kg for 7 days before inducing diabetes with intravenous STZ (50 mg/kg) [60]. HT treatments continued for 2 months following the induction of diabetes. Analysis of brain tissue showed that all concentrations of HT attenuated the damage caused by an experimental model of hypoxia and reoxygenation. Reductions in lipid peroxidation, nitrosative stress, cell death, and markers of brain inflammation (IL-1β and prostaglandin E_2_) were observed, showing a neuroprotective effect of HT against diabetes [60]. 

In another study, oral administration of HT (5 mg/kg) for 7 days before and 2 months after STZ injection resulted in reductions of diabetic retinopathy symptoms [61]. HT treatment attenuated the hyperglycemia-induced decrease in number of retinal ganglion cells, and increases in retinal thickness and retinal cell size [61]. Administration of HT (77 mg/kg/day) by intragastric gavage for 4 weeks decreased plasma glucose levels and markers of oxidative stress (nitric oxide (NO), malondialdehyde (MDA), while increasing levels of serum SOD and SIRT1 expression in the thoracic aorta and human umbilical vein endothelial cells (HUVEC) (Table 7) [62]. This study indicates that HT has some capacity to reduce oxidative stress and attenuate hyperglycemia and hyperlipidemia. 

### 3.3. Effect of Hydroxytyrosol (HT) on Genetically-Induced Diabetes in Rodents

Administration of HT by oral gavage (10 mg/kg/day) for 8 weeks to male hyperglycemic db/db C57BL/6J mice, lacking functional leptin receptors significantly reduced fasting glucose and lipid serum levels, in addition to reducing liver and muscle oxidative stress compared to nontreated control mice (Table 7) [63]. Metformin treatment also attenuated the increased fasting glucose but was not as effective as HT in controlling serum triglycerides and cholesterol levels [63]. In another study, administration of HT (10 or 50 mg/kg) daily for 8 weeks in male C57BL/6J db/db mice resulted in increased expression of mitochondrial respiratory chain complexes I/II/IV and the activity of complex I in the brain [64]. Additionally, HT significantly enhanced the activities of the antioxidant transcription factor p62 (sequestosome-1), haeme oxygenase 1 (HO-1) SOD 1, and SOD2, while attenuating protein oxidation in the brain. HT activated AMPK, SIRT1, and PPARγ-1α in brain tissue, demonstrating a possible neuroprotective effect against damage caused by high glucose levels [64]. 

### 3.4. Effect of Hydroxytyrosol (HT) on Diet-Induced Diabetes in Rodents

Administration of hydroxytyrosol-rich extract (3 mg/kg, respectively) for 17 weeks in Wistar rats fed a cholesterol-rich diet attenuated the increase in serum triglycerides, and total and LDL cholesterol. Additionally, serum antioxidant capacity and CAT, SOD enzymes in liver were significantly increased while TBARS were reduced in the liver, heart, kidney and aorta (Table 8) [65]. Administration of HT (10 or 50 mg/kg/day via gavage) for 17 weeks in obese and insulin resistant C57BL/6J mice reduced fasting glucose and insulin levels, fasting leptin, serum inflammatory markers IL-6 and CRP, serum triglycerides, and the serum LDL/HDL ratio [63]. No significant effect on serum adiponectin was observed. Hepatic and muscle tissue lipid content was decreased by HT, possibly explained by decreases in SREBP-1c and FAS expression [63]. HT also showed antioxidative capacity by decreasing oxidative damage to proteins and lipids in the liver and increasing hepatic GST and SOD enzyme activity [63]. Tabernero et al. (2014) used a cholesterol-rich diet to induce metabolic disease and hypercholesterolemia in male Wistar rats [66]. Over the course of 8 weeks, high-cholesterol (2%) diets were supplemented (0.04%) with HT, hydroxytyrosol-acetate (HT-Ac), or ethyl-hydroxytyrosol-ether (HT-Et) in the treatment group. All three compounds decreased plasma levels of insulin, leptin, MDA, total cholesterol, LDL cholesterol, glucose, and inflammatory markers TNFα and IL-1β in the hypercholesterolemic rats while increasing antioxidant capacity [66]. HT-Ac and HT-Et also decreased MCP-1 in visceral adipose tissue (VAT), which is associated with inflammation. HT-Ac treatment alone decreased plasma cholesterol levels, but the decrease seen in plasma free fatty acid (FFA) levels for all of the treatment groups were non-significant [66]. In another study, treatment of high-fat diet-induced diabetic mice with HT (20 mg/kg/day, orally for 3 weeks) resulted in reduced weight gain, visceral fat deposits, inguinal white adipose tissue (WAT) mass, and blood glucose levels [67]. Serum insulin and non-esterified fatty acids (NEFA) were not significantly changed by HT administration. Plasma mRNA levels of adiposity marker *Mest* was decreased in the treatment group compared to the control [67] (Table 8). Overall, HT demonstrated considerable potential in counteracting the effects of obesity and diabetes.

Pirozzi et al. (2016) administered a high-fat diet (58% fat) to male Sprague-Dawley rats for 6 weeks to induce non-alcoholic fatty liver disease (NAFLD), which starts with abnormal lipid accretion in hepatocytes (Table 8) [68]. The treatment group received 10 mg/kg of HT in addition to the high-fat diet via intragastric gavage. In the HT group, serum cholesterol and markers of liver damage (aspartate aminotransferase and alanine aminotransferase) were decreased while glucose tolerance and insulin sensitivity were improved [68]. Additionally, tissue assessments showed that HT inhibited hepatic inflammation (TNFα, IL-6, COX2), oxidative and nitrosative damage, and intestinal barrier damage. Carnitine palmitoyltransferase (CPT1a), PPARα, and acetyl CoA carboxylase (ACC) phosphorylation in the liver were all decreased by the high-fat diet, but this decrease was not observed in the treatment group, indicating the preservation of normal lipid metabolism in hepatic tissue by HT [68]. Administration of HT (20 mg/kg/day) for 8 weeks by oral gavage in high-fat, high-carbohydrate fed male Wistar rats showed attenuation of glucose intolerance, insulin resistance and weight gain [69]. Systolic blood pressure, heart inflammation, and liver damage were all reduced in the treatment group [69]. However, no effect was observed on plasma lipid levels. In this study, HT displayed protective effects on cardiac and hepatic tissue as well as improving glucose metabolism [69]. Administration of HT (20 mg/kg/day for 10 weeks) in high-fat diet-induced diabetic mice resulted in lower fasting glucose and insulin levels and increased GLUT4 expression in adipose and skeletal muscle tissue [70]. Modulation of insulin signaling in adipose tissue was shown by decreased serine phosphorylation of IRS-1 and increased Akt phosphorylation by HT [70]. Inflammation was decreased as indicated by lower levels of TNFα and IL-1β in the liver and adipose tissue as well as decreased serum CRP and IL-6 [70]. There were no significant changes in adiposity, serum adiponectin, serum lipids or liver (alanine aminotransferase (ALT), aspartate aminotransferase (AST) enzymes. However, HT was found to inhibit hepatic steatosis, lipid accumulation, and SREBP-1 mRNA expression in the liver; it also decreased indicators of endoplasmic reticulum stress in adipose tissue [70]. Similarly, oral administration of HT (5 mg/kg/day) to high-fat diet-induced diabetic mice for 12 weeks inhibited weight gain, hyperglycemia, insulin resistance, and the increases in hepatic and serum lipids caused by the high-fat diet. In the livers of the treated mice, steatosis scores were improved, anti-inflammatory markers eicosapentaenoic acid (EPA) and docosahexaenoic acid (DHA) were increased, and pro-inflammatory markers were decreased [71]. In this in vivo model of metabolic syndrome, HT demonstrated an ability to reduce hepatic inflammation and attenuate insulin resistance. 

In STZ and high-fat diet (HFD)-induced male diabetic Institute of Cancer Research (ICR) mice hydroxytyrosol-fenofibrate (FF-HT) (36 µmol/kg via intragastric gavage) for 11 weeks resulted in decreased plasma glucose, glucose intolerance, lipid profile (total cholesterol, triglycerides, LDL-cholesterol), atherosclerotic index (AI) and hepatic lipid accumulation compared to the fenofibrate control [72]. Additionally, antioxidant enzyme activities (SOD and, GSH-PX) were increased, while MDA and inflammatory markers TNFα and CRP were decreased. Furthermore, FF-HT treatment exhibited protective effects on pancreatic and hepatic tissues. The study also examined the effect of HT on Triton WR-1339 induced-hyperlipidemic mice further confirming the improvement in lipid profile (decrease in total cholesterol, triglycerides, LDL-cholesterol) and antioxidant activity (MDA decrease) [72]. Another study administered HT-nicotinamide (HT-N) or pure HT (both 0.38 mmol/kg by intragastric gavage) for 4 weeks in streptozotocin and high-fat diet (60 mg/kg for 7 days after 6 weeks of high-fat diet) - induced diabetic Kun Ming (KM) mice [73]. Results showed that both HT-N and HT caused a reduction in plasma glucose levels, total cholesterol, and triglycerides, while increasing the antioxidant activities of SOD, CAT, and GSH-PX. Additionally, treatment with both HT-N and HT showed a protective effect on pancreatic tissue and inhibited the destruction of β cells. The study also investigated the effect of HT on hyperlipidemic mice (induced with 400 mg/kg Triton WR-1339) showing decreased plasma triglyceride, cholesterol, and MDA levels [73]. In another study, Xie et al. (2018) investigated the effects of hydroxytyrosol-clofibrate (CF-HT, 240 µmol/kg/day for 7 days) in hyperlipidemic mice (induced with Triton WR-1339) showing decreased plasma total cholesterol and triglyceride levels, a protective effect on hepatic tissue (decreased AST, ALT, total bilirubin (TBIL), alkaline phosphatase (ALP)), and increased serum SOD and CAT. The CF-HT treatment additionally decreased serum MDA and hepatic oxidized glutathione (GSSH) while increasing hepatic glutathione (GSH) (Table 8) [74].

Numerous studies using experimental rodent models of diabetes have shown that HT can have beneficial in vivo effects against diabetes, obesity, and metabolic diseases. Diabetes was induced chemically with alloxan or streptozotocin, both of which damage β cells and inhibit production of insulin by the pancreas [75]. The studies using rodents with diabetes induced by alloxan or streptozotocin showed that HT may decrease serum glucose and lipid levels, mitigate inflammation, and significantly reduce oxidative stress. The harmful impact of hyperglycemia and lack of insulin production on the liver, heart, brain, pancreas, and retinal cells of the eye were also alleviated by HT [47,55,56,58,59,60,61,62,72,73,74]. Secondary experiments using Triton WR-1339 to chemically induce hyperlipidemia also showed that HT decreased serum lipids and liver damage [72,74]. HT has also been studied in genetic db/db C57BL/6J mouse models of diabetes, which lack leptin receptors and develop obesity and hyperglycemia [75]. Administration of HT to these mice was effective in reducing serum glucose and lipids as well as controlling oxidative stress in liver, muscle, and brain tissue [63,64]. Many in vivo studies researching HT have also used high-fat diets to induce metabolic syndrome in rodents. Most of these studies have reported that HT attenuated the increase in serum lipids, glucose, and insulin caused by the high-fat diets. They have also observed reduced oxidative damage, enhanced antioxidant capacity and decreased inflammation, with HT treatment [63,65,66,67,68,69,70,71]. These data suggest that HT has the potential to protect organs and tissues from damage caused by diabetes.

## 4. Effects of Hydroxytyrosol (HT) on Cellular Signaling Cascades

**Figure 2:**
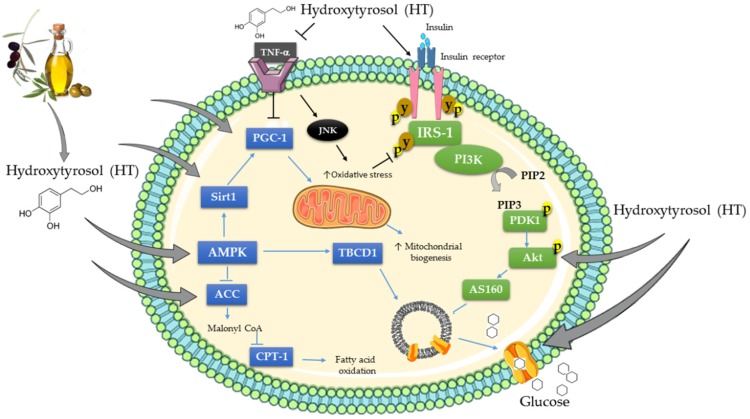
Effects of HT on cellular signaling molecules. The figure was created based on the evidence of the studies [33,37,43,64,70,76].

At the molecular/cellular level, HT was shown to increase glucose uptake in both muscle [31] and fat [70] cells. HT phosphorylated/activated the energy sensor AMPK and its downstream effector ACC in fat [33] and liver [43] cells, and increased the expression of SIRT1 in adipocytes [40] (Figure 2). Furthermore, HT treatment increased the expression of PGC-1 in skeletal muscle [29] and adipocytes [33]. Mitochondrial biogenesis, oxygen consumption capacity, ATP production, and activity of complex I, II and V in muscle cells and adipocytes were all increased by HT treatment [29,32,33] (Figure 2). Furthermore, treatment with HT increased the levels of adipose tissue phosphorylated/activated Akt, a crucial protein involved in insulin signaling [70]. 

HT reduced oxidative stress in muscle [30,63], liver [42,44,46,47,55,63,68,74], pancreas [56], kidney [65], heart [65], and brain tissue [60,64], as shown by the decreased levels of markers of oxidative stress such as NO and MDA and the increased activity of antioxidant enzymes including SOD, CAT, GSH and GPX. Additionally, HT reduced the inflammatory effects of TNF-α, and inhibited downstream the activation of JNK in adipocytes [37]. 

HT had significant anti-inflammatory effects. Serum CRP, IL-1β, IL-6 and TNF-α [55,58,59,63,66,70,71], liver TBARS, IL-1β, IL-6 and TNF-α [47,55,68], adipose IL-1β and TNF-α [70], pancreatic TBARS, AGE, LDH, CRP and IL-1β [56], and heart and kidney IL-6, TBARS levels were all reduced by HT treatment [65]. 

## 5. Summary, Conclusion and Future Directions

Existing studies indicate that hydroxytyrosol (HT) has insulin-like effects on insulin target cells including adipocytes, hepatocytes and muscle cells, and exerted significant anti-diabetic effects in animal models of T2DM. Moreover, HT exhibited protective effects against oxidative stress, inflammation, hyperglycemia and hyperlipidemia in chemically-, genetically- and dietary-induced animal models of T2DM. 

Although the current literature regarding HT toxicity is scarce, the evidence suggests that HT is well-tolerated. A study showed that oral administration of pure HT at 5, 50 and 500 mg/kg/day for 13 weeks in Wistar rats resulted in no adverse effects, including micro or macro organ alterations, morbidity, or mortality [77]. More importantly, in 2011, the European Food Safety Authority (EFSA) approved the use of HT (5 mg/day) or its derivatives to provide protection against oxidative damage and inflammation and reduce the risk of cardiovascular disease and insulin resistance/diabetes [78]. However, more systematic long-term human studies are required to assess the optimal dose required for the health benefits while avoiding any potential toxicity/side effects. In addition, more clinical studies are required to examine the bioavailability and mechanism of action of HT and to fully understand its antioxidant, anti-inflammatory and antidiabetic effects.

Overall, the existing in vitro and in vivo studies of HT showed potent antioxidant, anti-inflammatory, insulin-like, and insulin sensitizing effects. This suggests a potential application for HT in the prevention and treatment of insulin resistance and T2DM.

## Figures and Tables

**Table 1:** Most abundant phenolic compounds found in olive oil.

Polyphenol	Quantity	Olive Oil Type	Source
Hydroxytyrosol (3,4-dihydroxyphenyl ethanol) (HT)	0.93–14.64 mg/kg	Olive oil (various brands)	[21]
Tyrosol	0.25–14.97 mg/kg	Olive oil (various brands)	[21]
Oleuropein	0.0–4.7 mg/kg	Virgin olive oil	[22]

**Table 2:** Current pharmacological treatments for T2DM.

Antidiabetic Agent	Target Tissues	Target Pathways	Effect	Side Effects
Biguanides *metformin*	Liver, fat, muscle	↑ AMPK activity ↓ Complex I of the respiratory chain	↑ glucose uptake (fat and muscle) ↓ hepatic glucose production ↑ glucose tolerance ↑ insulin sensitivity	lactic acidosis GI problems (camps, nausea, vomiting, diarrhea)
Thiazolidinediones (TZD) glitazones: *rosiglitazone* *rioglitazone*	Liver, fat, muscle	↑ PPARγ activity	↑ adipocyte lipid storage ↓ circulating FFA ↓ ectopic lipid accumulation ↑ glucose uptake (fat and muscle) ↑ insulin sensitivity ↑ β-cell function	bladder cancer heart failure hepatitis bone fractures weight gain edema
Sulfonylureas glinides: *glyburide, glipizide* *glinepiride* meglitinides: *repaglinide*	pancreas fat, muscle	↑ intracellular potassium concentration leading to depolarization of pancreatic β cells	↑ glucose- mediated insulin release ↓ blood glucose	hypoglycemia weight gain hunger skin reactions
α-glucosidase-inhibitors: *acarbose, voglibose, miglitol*	small intestine	Competitive inhibition of enzymes vital for carbohydrate digestion	↓ carbohydrate absorption ↓ blood glucose	abdominal pain bloating, nausea, vomiting diarrhea flatulence
Dipeptidyl peptide 4 (DDP-4) inhibitors) gliptins: *sitagliptin, saxagliptin* *vidagliptin, linagliptin* *alogliptin*	pancreas	↓ DDP-4 activity ↑ incretin levels (GLP-1 and GIP)	↓ glucagon secretion ↑ insulin release ↓ blood glucose	heart failure pancreatitis pancreatic cancer prostate cancer GI problems flu-like symptoms
Incretin mimetics (glucagon-like peptide 1 (GLP-1) receptor agonist *liraglutide, exenatide* *dulaglutide* Gastric inhibitory polypeptide (GIP)	direct effect on pancreas, stomach and brain indirect on liver and muscle	↑ activation of GLP-1 receptor	↓ glucagon secretion ↑ insulin release ↓ β-cell apoptosis ↓ gastric emptying ↓ appetite ↓ blood glucose	pancreatic cancer pancreatitis heart failure prostate cancer abdominal pain nausea, vomiting
Sodium–glucose cotransporter 2 (SGLT2) inhibitors gliflozins: *canagliflozin* *capagliflozin*	kidneys	↓ SGLT2 action in the proximal convoluted tubule	↓ reabsorption of glucose ↑facilitate excretion in urine	Hypotension urinary tract infections ketoacidosis hyperkalemia kidney failure bone fractures

**Table 3:** Effects of hydroxytyrosol on skeletal muscle cells.

Cell Type	Hydroxytyrosol Concentration/Duration	Effect	Source
C2C12 myoblasts	1–50 µM for 30 min; TNFα for 4–5 days	↑ muscle cell differentiation (↑ creatine kinase & myosin heavy chain) ↑ PGC1α ↑ mitochondrial biogenesis	[29]
C2C12 myoblasts	5 or 20 µM HT for 3 h with 1 mM H_2_O_2_	↓ H_2_O_2_-induced apoptosis ↓ morphology changes ↓ oxidative stress	[30]
C2C12 myotubes	Hydroxytyrosol-acetate 0–75 µM for 12 h	↑ glucose uptake	[31]
C2C12 myotubes	1–50 µM for 24 h; 100 µM t-BHP	↑ cell viability ↓ mitochondrial dysfunction (↑ ATP production and activity of complex I, II and V) ↓ muscle cell degeneration (OPA cleavage) ↑ myosin heavy chain expression	[32]

**Table 4:** Effects of hydroxytyrosol on adipocytes.

Cell Type	Hydroxytyrosol Concentration/Duration	Effect	Source
3T3-L1 adipocytes	0.1–10 µM for 24–72 h	↑ mitochondrial biogenesis and O_2_ consumption ↑ mitochondrial complexes I, II, III and V ↓ fatty acid content ↑ CPT-1, PPARα, PPARγ	[33]
C3H10 T1/T2 preadipocytes	25 µM for 4 or 7 days	↓ lipid differentiation and accumulation ↓ lipid droplet size and number ↓ adipogenesis-related genes (PPARγ and C/EBPα) ↓ differentiation markers (aP2 and adiponectin) ↓ GLUT4 gene expression	[34]
3T3-L1 preadipocytes	100 or 150 µM for 0–8 days	↓ cell division and lipid accumulation ↓ mitotic clonal expansion ↓ adipogenesis marker genes (PPARg, SREBP-1c, C/EBPα, GLUT4, CD36 and FAS)	[35]
3T3-L1 adipocytes	Hydroxytyrosol-acetate 25–75 µM for 12 h	↓ lipid accumulation ↓ adipogenesis (PPARγ, SREBP-1c, C/EBPα, GLUT4, CD36, and FAS) ↑lipolysis and glycerol release ↑ HSL	[31]
3T3-L1 adipocytes	0–150 µM for 24–72 h	↑ lipolysis and glycerol release ↓ triglyceride accumulation ↑ HSL, ERK, perilipin phosphorylation ↓ ATGL, HSL, C/EBPα	[36]
3T3-L1 adipocytes SGBS adipocytes	0.1–20 µM with 10 ng/mL TNFα	↓ adiponectin suppression ↓ PPARγ suppression ↓ JNK phosphorylation	[37]
3T3-L1 preadipocytes	10–100 µM for 24–48 h 24 h only	↓ cell proliferation ↓ CBI receptor ↑ FAS, lipoprotein lipase (LPS) ↓ PPARγ	[38]
Primary human visceral preadipocytes	5–70 µg/mL for 20 days	↓ triglyceride accumulation ↑ apoptosis, lipolysis, glycerol release ↑ adipogenesis inhibition markers (GATA2, GATA3, WNT3A, SFRP5, HES1, and SIRT1) ↓ adipogenesis promoting genes (LEP, FGF1, CCND1, and SREBF1)	[40]
Human bone marrow MSC adipocytes	1 or 100 µM for 7–14 days	↑ adipogenesis markers PPARγ, FABP4 ↑ fat vesicle formation ↓ LPS	[41]

**Table 5:** Effects of hydroxytyrosol on hepatocytes.

Cell Type	Hydroxytyrosol Concentration/Duration	Effect	Source
Mouse hepatocytes	100 µM for 4 h (hypoxia); followed by reoxygenation	↓ cell apoptosis ↑ hepatocyte viability ↑ SOD1, SOD2, CAT activity	[42]
Rat hepatocytes	25 µM for 2 h	↓ lipid synthesis (fatty acid, cholesterol and triglyceride) ↓ ACC, diacylglycerol acyltransferase, 3-hydroxy-3-methyl-glutaryl-CoA reductase ↑ AMPK and ACC phosphorylation	[43]
Vit. E-deficient rat liver microsomes	0.05–2 mM for 30 min	↓ lipid peroxidation, TBARS	[44]
Vit. E-deficient rat liver microsomes	0.05–0.25 mM for 20 min	↓ lipid peroxidation, TBARS	[46]

**Table 6:** Effects of hydroxytyrosol on pancreatic cells.

Cell Type	Hydroxytyrosol Concentration/Duration	Effect	Source
Rat pancreatic tissue	50 µg/mL for 0–40 min with 4 g/L glucose	↓ decline in insulin secretion induced by hyperglycemia	[47]
Rat INS-1 β cells	0.1–30 µM 3-HT; 11 mM glucose for 1 h 3-HT:amylin ratio of 10:1 M for 0–40 h	↔ insulin secretion ↓ amylin amyloids	[48]

**Table 7:** Anti-diabetic Effects of Hydroxytyrosol: In vivo alloxan, streptozotocin- and genetic-induced diabetes animal studies.

Study Model	Hydroxytyrosol Concentration/Duration	Effect	Source
**Alloxan-Induced Diabetes Model**			
Alloxan-induced diabetic male Wistar rats	20 mg/kg for 2 months; intraperitoneal injection	↓ blood glucose levels ↓ liver TBARS, bilirubin, fatty cysts ↑ hepatic glycogen, HDL ↑ SOD, CAT, GPX in liver/kidney ↓ β cell damage	[47]
Alloxan-induced diabetic male Wistar rats	8 or 16 mg/kg orally for 4 weeks;	↓ blood glucose levels ↓ TC ↓ hepatic oxidative damage (TBARS) ↑ hepatic glycogen ↑ antioxidant enzymes (SOD, CAT)	[55]
**Streptozotocin-Induced Diabetes Model**			
STZ-induced diabetic male Wistar rats	20 mg/kg/day orally for 2 months	↓ blood glucose, HDL ↓ LDL cholesterol, TG ↓ intestinal enzymes (maltase, lactase and sucrose, lipase) ↑ pancreas SOD, CAT, GSH, GPX ↓ pancreas TBARS, AGE, LDH	[56]
STZ-induced male diabetic Sprague-Dawley rats	10 or 100 mg/kg/day for 6 weeks via gavage	↓ plasma TBARS ↑ NCV, thermal nociception Na+/K+-ATPase activity	[58]
STZ-induced male diabetic Wistar rats	0.5-10 mg/kg/day orally for 7 days prior to STZ and 2 months thereafter	↓ nitrosative, oxidative stress ↓ inflammation, IL-1β ↓ platelet aggregation ↓ aortic wall area	[59]
STZ-induced diabetic male Wistar rats	1, 5, or 10 mg/kg/day orally for 7 days prior to STZ and 2 months thereafter	↓ brain lipid peroxides, ↓ nitrosative stress, cell death ↓ brain inflammation, IL-1β, prostaglandin E_2_	[60]
STZ-induced diabetic male Wistar rats	5 mg/kg/day via endogastric cannula for 7 days prior to STZ and 2 months thereafter	↑ retinal ganglion cell number ↓ retinal thickness, cell size	[61]
STZ-induced diabetic KM mice	77 mg/kg/day HT or HT-NO via gavage for 4 weeks	↓ plasma glucose levels ↓ oxidative stress (↓ serum NO, MDA, ↑ SOD) ↑ SIRT1 expression in aorta and HUVEC)	[62]
**Genetic Diabetes Model**			
Male db/db C57BL/6J mice	10 mg/kg/day via gavage for 8 weeks	↓ fasting glucose levels ↓ serum lipids ↓ oxidative damage in liver and muscle	[63]
Male db/db C57BL/6J mice	10 or 50 mg/kg/day orally for 8 weeks	↑ mitochondrial complex I/II/IV expression (brain) ↑ activity complex I (brain) ↓ oxidative stress (↑ p62, HO-1) SOD1, SOD2) ↓ protein oxidation in brain ↑ AMPK, SIRT1, PPARγ -1α activation in brain	[64]

**Table 8:** Anti-diabetic Effects of Hydroxytyrosol: In vivo high-fat diet (HFD)-induced diabetes animal studies.

Study Model	Hydroxytyrosol Concentration/Duration	Effect	Source
Diet-induced hypercholesterolemic male Wistar rats	Olive leaf hydrolysate extract for 3 weeks (3 mg/kg b. w. orally containing HT (1.4 g/100 g dry weight), oleuropein	↓ serum TC, TG, LDL ↑ serum HDL ↓ TBARS (heart, liver, kidney) ↑ serum antioxidant capacity ↑ liver CAT, SOD activity	[65]
Diet-induced diabetic/obese male C57BL/6 mice	10 or 50 mg/kg/day via gavage for 17 weeks	↓ serum glucose, insulin ↓ serum IL-6, CRP, TG, leptin ↓ LDL/HDL ratio ↓ lipid content (liver, muscle) ↓ liver SREBP-1c, FAS ↓ liver protein carbonyls, MDA ↑ liver GST, SOD activity	[63]
Diet-induced hypercholesterolemic male Wistar rats	0.04% of diet with added HT, HT-Ac, or HT-Et for 8 weeks	↓ serum glucose, insulin ↓ serum leptin, MDA ↓ serum IL-1β, TNFα ↑ serum antioxidant activity ↓ VAT MCP-1, IL-1β ↓ serum TC, LDL (HT-Ac only)	[66]
Male C57BL/6J mice with diet-induced metabolic syndrome	20 mg/kg/day orally for 3 weeks	↓ serum glucose, *Mest* expression ↓ weight gain, visceral fat ↓ inguinal WAT ↔ serum insulin	[67]
Male Sprague-Dawley rats with diet-induced NAFLD	10 mg/kg/day via gavage for 6 weeks	↑ glucose tolerance ↓ serum glucose, insulin ↓ serum AST, ALT, TC ↑ hepatic PPARα, CPT1a, ACC ↓ liver TNFα, IL-6, COX-2 ↓ intestinal barrier damage ↓ liver ROS, MDA, RNS damage	[68]
Male Wistar rats with diet-induced metabolic syndrome	20 mg/kg/day via gavage for 8 weeks	↑ glucose tolerance ↓ serum insulin ↓ weight gain and fat mass ↓ liver steatosis, ventricular fibrosis ↓ plasma ALT, AST activity ↔ serum lipids	[69]
Diet-induced obese male ICR mice	20 mg/kg/day via gavage for 10 weeks	↓ fasting glucose, insulin ↑ glucose and insulin tolerance ↑ GLUT4 (adipocytes, myocytes) ↓ phospho-IRS-1 (Ser307) ↑ phospho-Akt (Ser473) ↓ liver, adipose tissue TNFα, IL-1β ↓ serum CRP, IL-6 ↓ hepatic steatosis, TG, ER stress ↓ liver SREBP-1 ↔ adiposity, adiponectin ↔ serum lipids, liver enzymes	[70]
Diet-induced obese male C57BL/6J mice	5 mg/kg/day orally for 12 weeks	↓ weight gain, insulin resistance ↓ serum glucose, insulin ↓ serum FFA, TAG, TC, LDL ↓ hepatic steatosis, FFA, TC ↓ liver TNFα, IL-1β/IL-6 ↑ hepatic EPA, DHA ↔ serum AST	[71]
STZ-induced diabetic male ICR miceTriton WR-1339 induced hyperlipidemic mice	36 µmol/kg/day FF-HT via gavage for 11 weeks 36 µmol/kg/day FF-HT via gavage for 7 days	↓ plasma glucose, lipids ↓ hepatic lipids ↓ TNFα, CRP ↑ glucose tolerance, antioxidants ↓ plasma TG, TC, MDA, atherosclerotic index (AI)	[72]
STZ-induced diabetic male KM mice Triton WR-1339 induced hyperlipidemic mice	0.38 mmol/kg/day via gavage for 4 weeks 0.38 mmol/kg/day via gavage for 7 days prior to Triton WR	↓ blood glucose, lipids ↑ plasma SOD, CAT, GSH-PX ↓ plasma TG, TC, MDA	[73]
Triton WR-1339 induced hyperlipidemic mice	240 µmol/kg/day for 7 days	↓ plasma TG, TC, MDA ↓ AST, ALT, TBIL, ALP, hepatic GSSH ↑ serum SOD, CAT ↑ hepatic GSH	[74]
